# Lymphatic Recovery and Clinical Implications in Hand Allotransplantation: Insights From Indocyanine Green Lymphangiography

**DOI:** 10.7759/cureus.102103

**Published:** 2026-01-22

**Authors:** Ramu Janarthanan, G Srilekha Reddy, Mohit Sharma, Kishore P, Jimmy Mathew, Sam Thomas, Vasundhara Jain, Sri Valli Vemulapalli, Fatih Zor, Yalcin Kulahci, Vijay S Gorantla, Subramania Iyer

**Affiliations:** 1 Plastic and Reconstructive Surgery, Amrita Institute of Medical Sciences, Amrita School of Medicine, Amrita Vishwa Vidyapeetham, Kochi, IND; 2 Wake Forest Institute for Regenerative Medicine, Wake Forest School of Medicine, Winston-Salem, USA; 3 Plastic and Reconstructive Surgery, Amrita Research Centre, Amrita Vishwa Vidyapeetham, Faridabad, IND; 4 Plastic and Reconstructive Surgery, Indiana University School of Medicine, Indianapolis, USA; 5 Department of Surgery, Advocate Health, Wake Forest School of Medicine, Winston-Salem, USA

**Keywords:** allograft rejection, hand transplant, indocyanine green lymphangiogram, lymphangiogenesis, upper extremity transplantation

## Abstract

Introduction

Hand vascularized composite allotransplantation (VCA) is currently considered a promising reconstructive approach for selected hand amputees. The major limitation of hand VCA is its risk of rejection, which influences the long-term survival of the allograft. The lymphatic system plays a key role in inflammation, antigen presentation, and immune regulation, yet lymphatic regeneration following upper limb VCA is poorly understood. The correlation between donor-recipient lymphatic reconnection and the allograft recovery and rejection is not well understood. This study was designed to describe lymphatic drainage patterns following upper limb transplantation using indocyanine green lymphangiography (ICG-L) and to explore descriptive associations between these patterns and clinical graft status.

Materials and methods

This single-center, descriptive case series included seven patients who underwent bilateral upper limb transplantation in the tertiary care facility. ICG-L was performed on one transplanted limb per patient at a single, randomly selected follow-up time point. Indocyanine green dye was injected intradermally at the first webspace, fourth webspace, and the ulnar aspect of the wrist on the flexor surface. The lymphatic flow was visualized using near-infrared fluorescence imaging. Lymphatic drainage patterns were assessed within the allograft, across the donor-recipient interface, and in the recipient limb, and were categorized as linear (normal) or non-linear (abnormal) using Yamamoto’s classification. Clinical data, including limb edema and biopsy-proven rejection episodes, were collected. All data were analyzed descriptively; no formal statistical correlation was performed.

Results

ICG-L was performed with timing varied from three months to four years and two months post-transplant. No adverse reactions to indocyanine green were observed. Six patients had no clinical lymphedema, while one patient demonstrated persistent edema associated with chronic rejection. A total of 21 rejection episodes were documented. Limbs demonstrating linear lymphatic flow at the donor-recipient interface accounted for 12 rejection episodes, while limbs with non-linear flow accounted for nine episodes. Lymphatic continuity into the recipient limb was observed in all but one supracondylar-level transplant.

Conclusion

Lymphatic regeneration occurs following upper limb VCA despite the absence of surgical lymphatic anastomosis. The observed lymphatic patterns may reflect the complex role of lymphatics in edema resolution and alloimmune responses. The findings in this study are exploratory, and they highlight the need for further research on lymphatic reconnection and its potential for improving allograft function and immunotolerance. Limitations include a small sample size and a lack of serial time-bound measurements.

## Introduction

Vascularized composite allotransplantation (VCA) of the hand has demonstrated meaningful functional and psychosocial benefits in upper limb amputees. VCA involves the transfer of multiple heterogeneous tissues, including skin, muscle, bone, vessels, nerves, and tendons, each with differing antigenic potential. Among these, skin represents the most immunogenic component and contains an extensive lymphatic network. The constant exposure of the skin to the external environment makes upper limb allografts particularly susceptible to inflammation and immune-mediated injury. The lymphatic system plays a fundamental role in tissue homeostasis, immune surveillance, and the resolution of inflammation. The lymphatics are essential for clearing inflammatory mediators and excess interstitial fluid, which aids in postoperative recovery. Concurrently, it also facilitates the transport of antigens and antigen-presenting cells to naïve T cells in the regional lymph nodes, contributing to adaptive immune responses [1].

Although lymphatic vessels are known to be critical in antigen presentation and immunoregulation, their role in allograft rejection and tolerance remains incompletely understood. Experimental and clinical studies have produced conflicting evidence, with some suggesting that lymphatics may facilitate alloimmune responses, while others indicate a role in promoting immune regulation and graft survival. This apparent duality underscores the complexity of lymphatic involvement in transplantation biology. Despite the standard of care, hand VCA recipients experience at least one episode of acute rejection within the first year of transplantation [2], providing further rationale to investigate additional biological pathways, such as the lymphatic system, that may influence graft outcomes.

Consequently, evaluation of lymphatic regeneration and flow following upper limb transplantation is clinically relevant, particularly because lymphatic channels are not surgically reconnected during VCA procedures. Currently, there is a paucity of data on the clinical evaluation and imaging of lymphatic regeneration in hand VCA. Therefore, evaluation of lymphatic channels, their patterns of reestablishment, and their relationships with clinical parameters and rejection may provide important insights into the role of the lymphatic system in VCA and inform future hypothesis-driven research.

This work was presented as a poster at the International Society of Vascularized Composite Allotransplantation (ISVCA) Congress in June 2025 [3].

## Materials and methods

This study was designed as a descriptive, exploratory case series to characterize lymphatic drainage patterns following upper limb VCA using indocyanine green lymphangiography (ICG-L) and to qualitatively compare these patterns with clinical graft outcomes, including limb edema and biopsy-proven rejection episodes. The primary objective was to describe lymphatic drainage patterns in transplanted upper limbs using ICG-L. The secondary objective was to descriptively assess associations between lymphatic patterns and clinical findings, including limb edema and biopsy-proven acute or chronic rejection. This study aims to provide preliminary clinical observations on lymphatic regeneration in upper limb transplantation and to generate hypotheses for future studies investigating the immunological and functional relevance of lymphatic reconnection in VCA.

This is a single-center study evaluating lymphatic drainage following bilateral upper limb VCA in the tertiary care facility. Institutional Ethics Committee approval was obtained prior to study initiation (ECASM-AIMS-2025-422), and written informed consent was obtained from all participants. Given the rarity of the procedure and limited sample size, the study was designed as an exploratory, descriptive investigation without hypothesis testing or formal statistical inference. The correlation between lymphatic flow patterns and clinical data, including edema and rejection episodes, was descriptive and observational in nature.

Seven patients who underwent bilateral upper limb VCA at our institution were included. All patients were followed postoperatively according to institutional transplant protocols. Demographic and clinical data were obtained retrospectively from medical records. All patients received induction immunosuppression with antithymocyte globulin. Maintenance immunosuppression consisted of tacrolimus, mycophenolate mofetil, and prednisolone. Tacrolimus trough levels were maintained at 10-15 ng/mL during the first postoperative year and 5-10 ng/mL thereafter. Clinical variables included were skin changes, limb edema, episodes of acute rejection, and chronic rejection. Acute or chronic rejection was diagnosed clinically and confirmed histopathologically on skin biopsy specimens, graded according to the Banff criteria for VCA [4]. Rejection episodes were managed as per institutional protocol.

ICG-L was performed at a single follow-up visit in each patient on one transplanted upper limb, without side predilection. Indocyanine green was prepared by diluting 25 mg of lyophilized ICG (Aurogreen®, Aurolab, Tamil Nadu, India) in 10 mL of sterile distilled water. A volume of 0.2 mL was injected intradermally at the first web space, fourth web space, and ulnar aspect of the volar wrist. Lymphatic flow was visualized using an infrared fluorescence imaging system (Irillic, Bangalore, India) at 10 minutes, one hour, and two hours post-injection.

The lymphatic drainage patterns were categorized using Yamamoto’s classification system [5], which describes linear and non-linear lymphatic patterns. Non-linear patterns included splash, stardust, and diffuse appearances. For the purpose of analysis in this study, lymphatic patterns were broadly grouped as follows: linear flow (indicative of functional lymphatic drainage) and non-linear flow (indicative of impaired or abnormal lymphatic function).

All data were analyzed descriptively with the qualitative comparison of lymphatic drainage patterns observed on ICG-L with documented clinical findings, including limb edema and biopsy-proven acute rejection episodes.

## Results

Seven patients were included in the study, comprising six males and one female, with ages ranging from 19 to 34 years. The most common etiology of limb loss was electrical injury (n = 4), followed by crush injuries sustained in train or bus accidents (n = 2), and blast injury (n = 1). Demographic and clinical details are summarized in Table 1.

**Table 1:** Clinical details of the patients.

Case No.	Age/Sex	Etiology	Level of Transplant	Time ICG-L (Post-transplant)	Clinical status of limb during ICG-L	ICG Lymphangiogram - Flow Patterns			Rejection Episodes		Follow-up
						Donor Limb (Allograft)	Donor-Recipient Interface	Recipient Limb	Pre ICG-L	Post ICG-L	
1	30/M	Train accident	Distal forearm	4 years and 2 months	No edema skin normal	Linear flow	Linear flow, patchy area of Nonlinear flow	Linear flow	5	0	9 years and 8 months
2	31/M	Bomb blast	Distal forearm	4 years	No edema skin normal	Linear flow	Linear flow	Linear flow	3	0	9 years and 5 months
3	21/M	Electrical injury	Proximal forearm	2 years and 10 months	Edema + skin changes + chronic rejection +	Linear flow	Nonlinear flow	Linear flow	5, later developed chronic rejection	Chronic rejection	8 years
4	19/F	Road traffic accident (crush injury)	Supracondylar	1 year and 5 months	No edema skin normal	Linear flow progressing into Nonlinear flow	Nonlinear flow	Nonlinear flow progress to Linear flow	2	1	7 years and 2 months
5	34/M	Electrical injury	Proximal forearm	5 months	No edema skin normal	Linear flow progressing to Nonlinear flow in proximal forearm and elbow	Nonlinear flow	Linear flow - irregular	1	0	3 years and 2 months
6	24/M	Electrical injury	Supracondylar	3 months	Mild edema for 1 year skin normal	Linear flow	Linear flow predominant on extensor side, Nonlinear flow on flexor side	Non visualization	0	2	2 years and 10 months
7	28/M	Electrical injury	Supracondylar	12 months	No edema skin normal	Linear flow, patchy areas of Nonlinear flow in proximal forearm	Linear flow	Linear flow	0	2	2 years and 8 months

Regarding the level of transplantation, two patients underwent bilateral distal forearm transplantation, two underwent bilateral proximal forearm transplantation, and three underwent bilateral supracondylar transplantation. ICG-L was performed on the right upper limb in four patients and the left upper limb in three patients. The timing of ICG-L following transplantation ranged from three months to four years and two months, with a mean interval of 14 months. No allergic or hypersensitivity reactions to indocyanine green were observed. Patients experienced mild pain at the injection sites, which resolved within two days. One patient developed mild limb edema following the procedure, which resolved within one week with compression bandaging.

**Figure 1:**
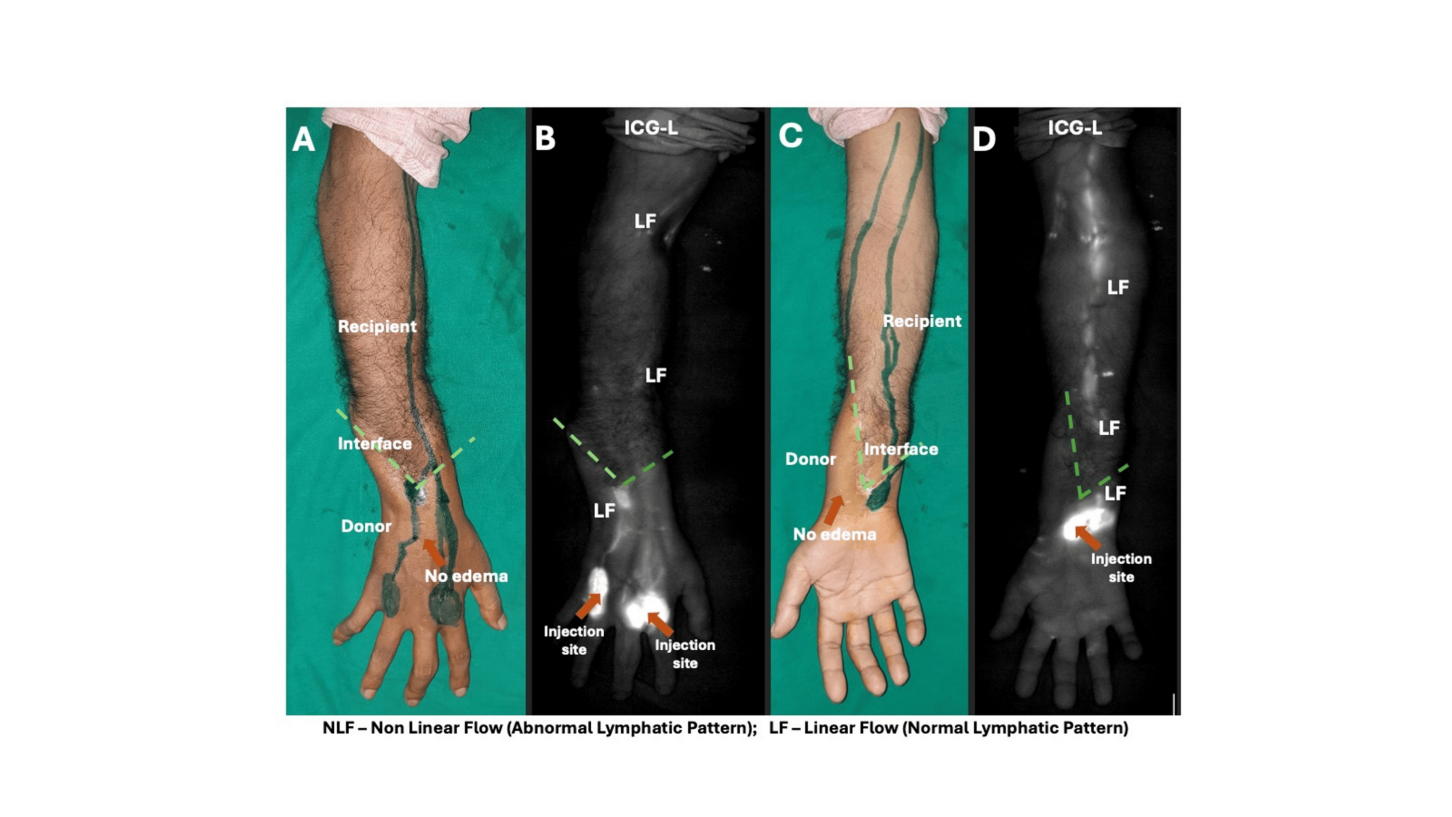
Clinical image showing distal hand transplant with no edema in case 1 (A, C). ICG-L shows linear pattern lymphatic flow in the allograft, interface, and the recipient limb (B, D).

**Figure 2:**
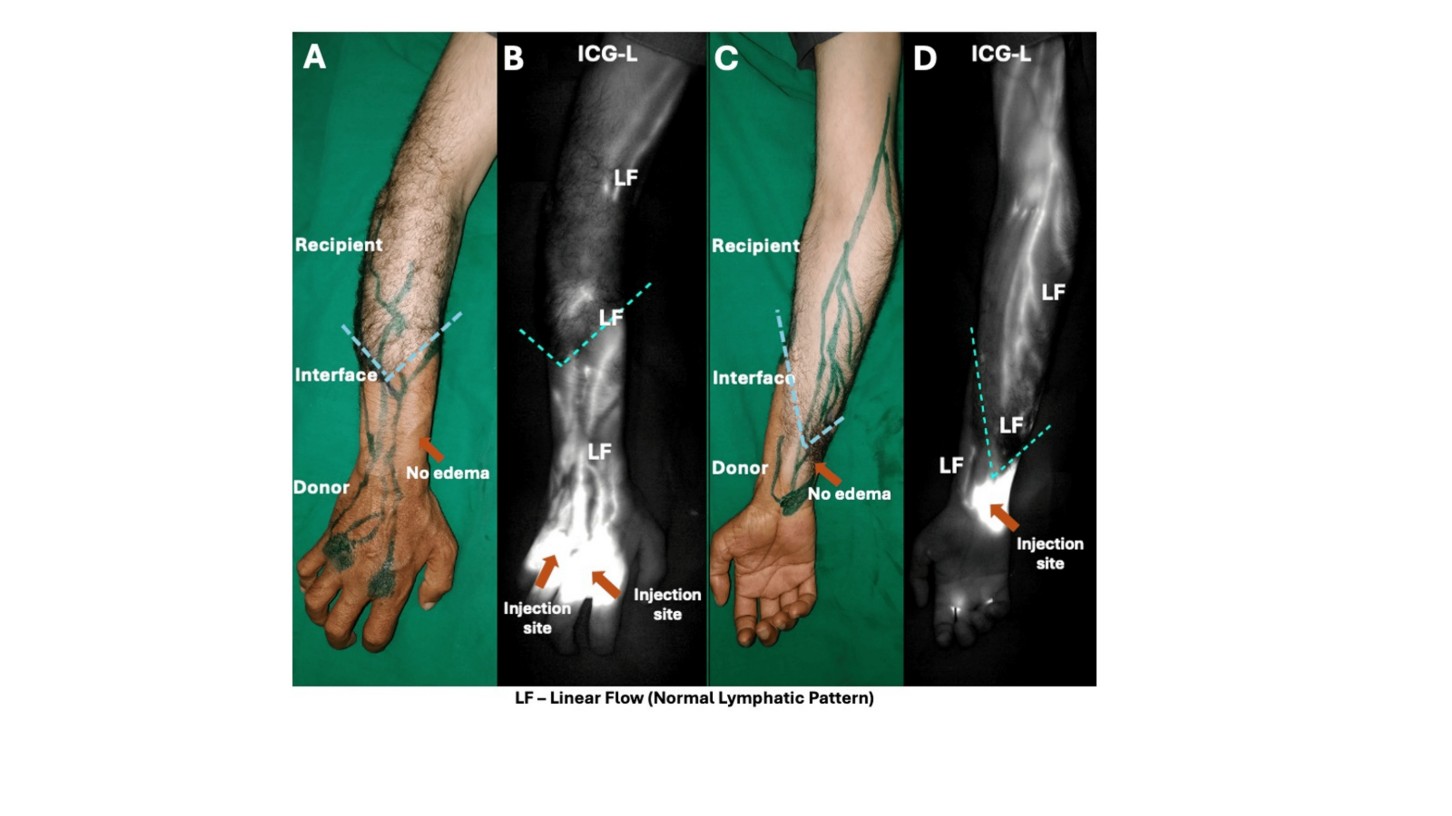
Clinical image showing no edema and normal skin in case 2 (A, C). ICG-L showing multiple linear lymphatic channels in the allograft, interface and the recipient limb (B, D).

**Figure 3:**
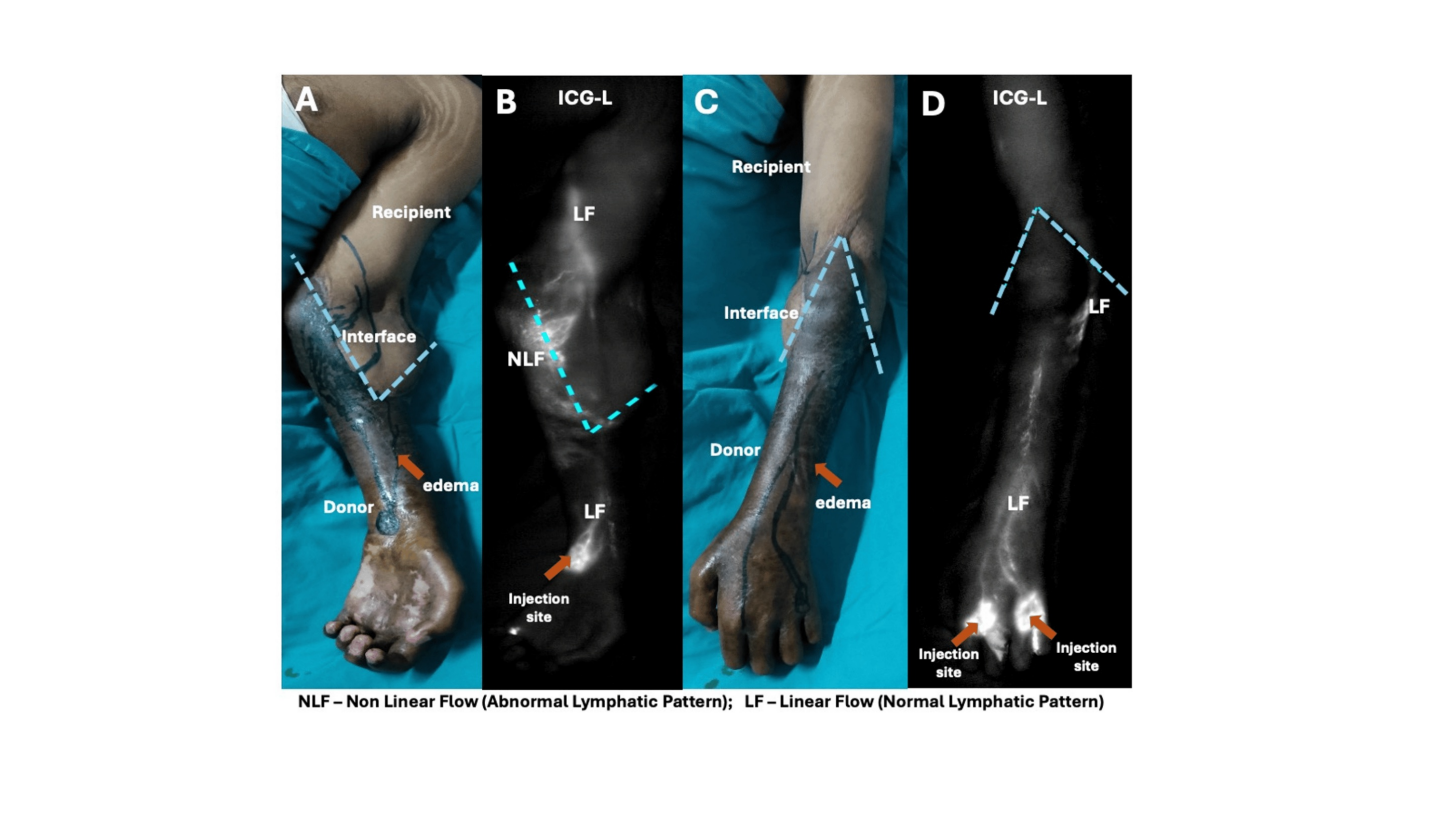
Developed signs of chronic rejection at 2 years and 5 months following transplant in case 3. Skin changes, nail changes, and edema could be observed in the clinical picture (A, C). ICG-L showing linear pattern of flow in the allograft and recipient limb with nonlinear pattern in the interface (B, D).

**Figure 4:**
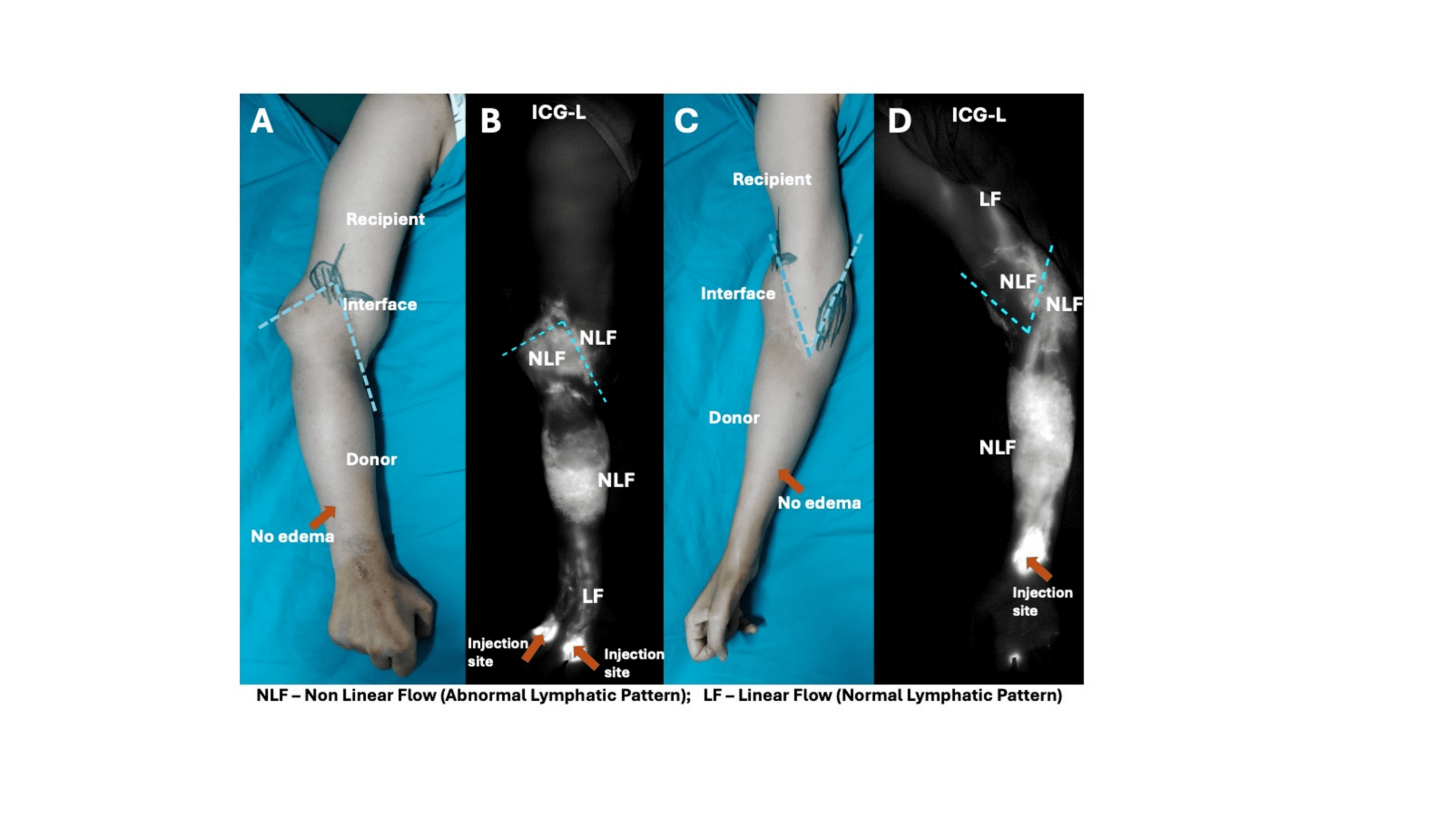
Young female with supracondylar transplantation, showing no signs of clinical edema (A, C) despite the ICG-L showing abnormal nonlinear patterns in the allograft and interface in case 4 (B, D).

All patients exhibited edema predominantly within the allograft during the immediate postoperative period, with more severe edema observed in proximal-level transplants. In distal forearm transplants, edema resolved within four to eight weeks, whereas in proximal-level transplants, resolution required up to six months. Representative ICG-L images at different levels of upper limb transplantation are shown in Figures 1-4. While overall flow within the donor allograft was linear in all patients, focal areas of non-linear flow were observed in three, particularly near the donor-recipient interface. On the recipient limb side, four patients demonstrated linear lymphatic flow patterns. In patients with distal forearm transplantation, linear lymphatic flow was well established within the allograft, across the interface, and into the recipient limb in both cases, with one patient demonstrating a focal area of non-linear flow at the interface. Among proximal forearm transplant recipients, non-linear patterns were observed at the interface; however, lymphatic flow within both the allograft and recipient limb was predominantly linear. In supracondylar transplant recipients, linear lymphatic flow was observed within the allograft and interface in all cases, while only one patient demonstrated linear flow across the recipient limb.

All patients experienced at least one episode of acute rejection following transplantation, with a total of 21 biopsy-proven rejection episodes recorded across the cohort. Limb demonstrating linear lymphatic flow at the donor-recipient interface were associated with 12 rejection episodes, while limbs demonstrating non-linear flow patterns at the interface were associated with nine rejection episodes. The patient who had chronic rejection developed persistent edema in the allograft.

ICG-L images of representative cases at different levels of hand transplantation are shown in Figures 1-4. All patients had a predominantly linear pattern of lymphatic flow in the donor side (allograft), with three patients showing nonlinear flow as traced towards the interface. At the donor-recipient interface, three among seven patients showed linear flow across the allograft. On the recipient limb side, four patients showed linear flow. In the two distal transplant patients, the normal linear flow was well established in the donor limb, interface, and recipient limb, except for a patchy area of a nonlinear pattern in the interface in one patient. Among the two proximal forearm cases, although abnormal flow patterns were noticed in the interface, the allograft and recipient side showed a linear pattern predominantly. In three cases of supracondylar transplants, a predominant linear pattern was noticed in the allograft and interface; only one showed a good linear pattern in the recipient limb. Axiality of lymphatics was noted in three limbs. Patients had a total of 21 rejections following transplantation, with at least one rejection in each patient. The limbs showing linear and nonlinear (abnormal patterns) lymph flow at the donor-recipient interface had a total of 12 and nine rejections, respectively.

## Discussion

In this exploratory case series, we used ICG-L to characterize postoperative lymphatic drainage patterns following upper limb VCA and to qualitatively relate these patterns to clinical outcomes. Our findings demonstrate marked inter-recipient variability in lymphatic reestablishment, with differences in drainage patterns corresponding to the severity and persistence of postoperative limb edema and the occurrence of biopsy-proven rejection episodes. Given that lymphatic channels are not surgically reconnected during hand VCA, these observations suggest that the rate and pattern of lymphangiogenesis may influence both fluid homeostasis and local immune activity within the graft. The observed variability in lymphatic function highlights the potential clinical relevance of lymphatic regeneration in VCA and supports further investigation into its role in graft inflammation, edema, and alloimmune responses.

Disruption of lymphatic function can exacerbate inflammation and potentially influence alloimmune responses [6]. This underscores the significance of the lymphatic system of the skin in mitigating inflammatory reactions. Restoration of lymphatic continuity between donor and recipient tissues following hand VCA relies entirely on spontaneous regeneration to reestablish lymphatic flow. Previous studies in flap transfer and limb replantation have demonstrated that lymphatic regeneration and functional recovery are possible [7-9]; however, data specific to clinical upper extremity VCA remain limited. The rate and extent of lymphatic regeneration are species- and organ-specific, with reported onset ranging from as early as three days to as late as 14 days after surgical disruption [7,8,10]. Despite this variability, functional lymphatic reconnection is generally achieved by approximately two weeks postoperatively. In a non-human primate VCA model, the timing of lymphatic reconnection was correlated with a reduction in postoperative swelling [11]. There are no studies related to the timing of lymphangiogenesis following clinical VCA. Considering the fact that immunosuppressive agents used in hand VCA may also influence lymphatic regeneration and function, although their effects remain incompletely understood. Tacrolimus, mycophenolate mofetil, and corticosteroids have been shown to exert variable effects on lymphatic endothelial cells and inflammatory pathways in experimental and clinical models [12-16]. In this study, lymphatic reconnection was observed despite standard immunosuppressive therapy. However, the study design precludes conclusions regarding the timing of lymphangiogenesis or the impact of specific immunosuppressive agents on lymphangiogenesis and lymphatic function. Nevertheless, postoperative lymphatic function varied among recipients, resulting in differing degrees of lymphatic dysfunction.

**Figure 5:**
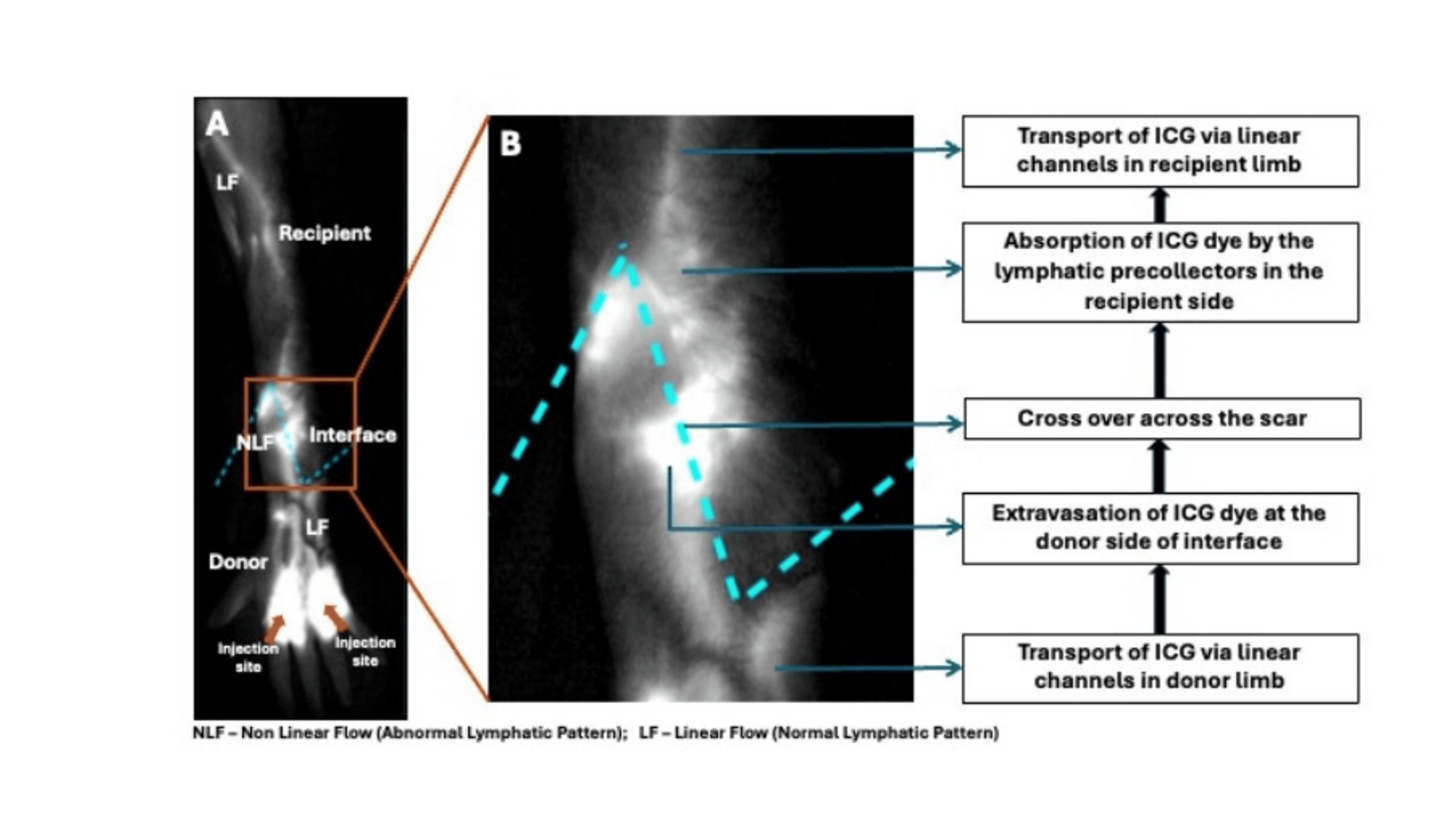
Potential mechanism of lymphatic flow across the donor-recipient interface following hand transplantation in the absence of lymphatic recanalization (A, B).

Edema is a clinically relevant manifestation of lymphatic dysfunction and is commonly observed following hand transplantation. Postoperative edema in hand VCA is multifactorial and not solely attributable to impaired lymphatic drainage; contributing factors are summarized in Table 2. In our cohort, distal forearm transplants were associated with less pronounced and shorter-duration edema compared with more proximal transplants, a finding that may be related to the larger tissue volume and increased lymph production in proximal-level allografts. During the immediate post-transplant period, lymph absorbed within the allograft likely accumulates and leaks at the donor-recipient interface, as lymphatic channels are not yet recanalized and drainage depends primarily on absorption by recipient lymphatic capillaries. As lymphatic regeneration progresses, lymphatic flow may be partially restored through recanalized donor and recipient lymphatic channels, while residual flow across the interface is likely mediated by lymphatic precollectors within the scar tissue (Figure 5). Failure of recanalization of major lymphatic channels may result in persistent dermal backflow, increased intraluminal pressure, progressive vessel and valvular dysfunction, and ongoing clinical edema. Our clinical experience suggests that postoperative edema typically subsides substantially within four to six weeks and continues to improve for up to six months. However, we did not observe clinically apparent edema during episodes of acute rejection, whereas one patient who developed chronic rejection exhibited persistent edema.

**Table 2:** Factors influencing post-surgical edema in hand transplant

Category	Factors
Recipient-related	Local scarring and disrupted lymphatic drainage at the amputation stump; level of amputation
Ischemia-reperfusion	Prolonged ischemia time; ischemia-reperfusion injury; reperfusion-related capillary leak
Intraoperative factors	Hemodynamic instability; large-volume fluid resuscitation and blood transfusion
Venous outflow	Limited number or quality of venous anastomoses; impaired venous drainage
Lymphatic factors	Severed lymphatics not reconstructed; disruption of lymphatic axiality during donor-recipient skin flap alignment
Neurogenic factor	Denervation of the allograft (impairs lymphatic and venous tone)
Inflammatory factors	Surgical site inflammation; infection; increased capillary permeability
Immunologic factors	Allograft rejection; need for immunosuppression intensification
Pharmacologic factors	Systemic and topical immunosuppressants; high-dose corticosteroids
Postoperative factors	Dependent limb positioning; immobilization; delayed physiotherapy

In this study, imaging showed that lymphatic flow across the donor-recipient interface into the recipient limb was observed in all but one patient. Predominantly linear lymphatic flow was noted within the donor allograft, consistent with the presence of intact donor lymphatic channels. In contrast, non-linear lymphatic flow patterns were frequently observed near the donor-recipient interface, likely reflecting the regeneration of the disrupted lymphatics at the interface without forming a linear channel. In the absence of direct lymphatic anastomosis, lymphatic flow across the interface is likely mediated by newly formed lymphatic channels or by absorption through lymphatic capillaries and precollectors within the scar tissue. Some of these clinical and imaging findings were consistent with previous preclinical and clinical VCA studies [10,11,17]. Notably, Cavadas et al. reported moderate edema in two of three patients following hand transplantation using lymphoscintigraphy [17]. However, these observations should be interpreted descriptively, with consideration of the multifactorial nature of edema.

Clinical experience with extremity lymphedema has demonstrated an increased risk of recurrent infections and chronic inflammation due to impaired lymphatic function and inadequate clearance of pathogens [18]. Inflammation is a key underlying mechanism in both acute and chronic rejection of an allograft. Lymphatic dysfunction-induced inflammation may therefore contribute to allograft rejection [19], while strategies aimed at alleviating inflammation may help reduce rejection risk [20]. There is growing interest in leveraging lymphangiogenesis and the lymphatic system for immunomodulation in VCA [21,22]. However, the literature provides conflicting evidence regarding the role of the lymphatic network in regulating immune responses to allografts. Recently, vascular endothelial growth factor (VEGF) has been identified as one of the key regulators in this process [23-26]. Modulation of these factors has therefore been proposed as a potential strategy to either enhance or suppress immune responses in transplantation. However, the key question is to determine its role in allograft function and rejection.

Experimental studies have suggested that lymphangiogenesis may exacerbate allograft rejection by facilitating antigen-presenting cell trafficking from the graft to regional lymph nodes, thereby amplifying alloimmune activation [27-30]. Inhibition of it has been shown to improve graft survival in kidney, corneal, liver, and pancreatic islet transplantation models [31-33]. Conversely, other studies demonstrate a protective or tolerogenic role for lymphatic regeneration, with enhanced lymphangiogenesis associated with improved graft survival in murine kidney and lung transplantation models [34,35]. Clinical studies further support this concept, showing that higher lymphatic vessel density in human kidney and cardiac allografts correlates with lower rejection rates and improved functional outcomes [36,37]. Impaired lymphatic drainage has also been linked to tertiary lymphoid organ formation (TLO) and graft dysfunction in lung transplantation [38], while acute rejection has been associated with cessation of lymphatic drainage in canine lung allografts [39].

In the context of these divergent findings, our results underscore the complexity of lymphatic-immune interactions in hand VCA. Although all patients experienced at least one rejection episode irrespective of lymphatic flow pattern, we observed that some patients with established linear lymphatic flow demonstrated stable graft function on longer follow-up. However, given the descriptive nature of our study and the absence of longitudinal lymphatic imaging, these findings cannot establish the exact role of lymphangiogenesis. It is plausible that recurrent rejection episodes may themselves impair lymphatic integrity, leading to reduced clearance of immune cells and sustained inflammation, thereby perpetuating further rejection. Collectively, our observations align with the broader literature suggesting that lymphangiogenesis may exert context-dependent effects on allograft immunity, highlighting the need for further longitudinal, mechanistic studies to better define its role in hand VCA. Thus, future studies focusing on lymphatic regeneration, therapeutic lymphangiogenesis, and the reconnection of lymphatics in VCA will expand our understanding of the role of lymphatics in transplant immunology and how to leverage this knowledge to improve allograft survival.

The primary limitations of this study include its descriptive observational study design with a small sample size and the absence of formal statistical analysis. Lymphatic imaging was performed at a single, non-standardized time point, preventing assessment of temporal changes in lymphatic regeneration. The analyses were descriptive and not designed to establish causal relationships between lymphatic patterns and rejection. These factors limit inferential conclusions; however, they are inherent to investigations involving rare procedures such as upper limb VCA. Despite these limitations, the study provides clinical observations that may guide future hypothesis-driven research.

## Conclusions

Lymphatic networks are among the least explored pathways for immunomodulation in VCA. The prevention of allograft rejection remains a challenging issue, despite the current standard of care in VCA. Therefore, it is imperative to investigate additional strategies that could synergistically improve allograft survival. With this study limitation of small number of cases and difficulty in clinically directing the study focusing only on lymphatics and immune system, their role in reducing postoperative allograft edema is well noted in this study. The observed variability in lymphatic flow patterns and their coexistence with rejection episodes highlight the complex and context-dependent role of the lymphatic system in allograft biology. These observations do not establish causality but suggest that lymphatics may participate in both immune activation and immune regulation, depending upon the context. Future prospective studies with larger cohorts and longitudinal lymphatic imaging are needed to clarify the immunological significance of lymphatic reconnection in VCA. Further investigation into therapeutic lymphangiogenesis and selective lymphatic reconstruction may help determine whether targeted modulation of the lymphatic system can enhance allograft function, reduce edema, and potentially contribute to improved immunological outcomes.
